# CaOx crystal nuclei are formed in rat outer cortex proximal tubules by a potential fibrinogen-dependent mechanism

**DOI:** 10.1371/journal.pone.0328721

**Published:** 2025-09-09

**Authors:** Kenshirou Kunii, Shigeru Sugiki, Chiharuko Ushimoto, Shinya Inoue, Nobuyo Morita, Yuka Nakamura, Tetsuhiro Horie, Takuya Sakamoto, Hiromi Sakata-Haga, Jia Han, Toshihisa Hatta, Yasuhito Ishigaki, Sohsuke Yamada, Katsuhito Miyazawa

**Affiliations:** 1 Department of Urology, Kanazawa Medical University, Kahoku, Ishikawa, Japan; 2 Medical Research Institute, Kanazawa Medical University, Kahoku, Ishikawa, Japan; 3 Department of Anatomy I, Kanazawa Medical University, Kahoku, Ishikawa, Japan; 4 Department of Clinical Pathology, Kanazawa Medical University, Kahoku, Ishikawa, Japan; Universidade de Sao Paulo, BRAZIL

## Abstract

Calcium oxalate (CaOx) stones are prevalent in urinary tract stone disease. While their formation can be induced in rats by administering ethylene glycol and vitamin D, the initial nucleation and formation processes are unclear. Here, we aimed to determine where CaOx crystals initially form, examine the associated histological and morphological changes, and clarify the genes whose expression varies at those sites and their function. Male Wistar rats were divided into four groups: control, ethylene glycol, vitamin D, and ethylene glycol plus vitamin D (EG + VitD). Crystal development locations were mapped on kidney tissue sections, and the initial crystal site distribution was revealed. CaOx crystal formation was observed only in the EG + VitD group kidneys, predominantly in the proximal tubules in the outer renal cortex. The tubular luminal area was significantly increased (*P* < 0.05), especially in proximal tubules, correlating with the crystal occurrence number. Moreover, aquaporin1 and calbindin staining identified the tubular segments hosting initial crystal formation, and the tubular dilation was calculated. DNA microarray was analyzed on cortical and medullary kidney tissues to detect stone formation-related gene expression changes. Genes with variable expression were further examined using RT-PCR and immunohistochemistry to analyze their distribution. *FGA, Slc7a9, Slc7a7, and TRPV5* were significantly upregulated in the renal cortex, and *FGA* was significantly upregulated in the proximal tubules, consistent with the crystal formation sites. Early phase crystallization primarily occurs in the proximal tubules. In silico analysis, FGA protein has multiple oxalic acid-binding sites, making it a potential new factor promoting CaOx crystal formation.

## Introduction

The models used for urinary tract stone research have primarily been laboratory animals and cultured cells; however, since Randall’s plaques have been identified and with the advances in medical technology, the research methods have changed. In fact, some reports have investigated the pathogenesis of stone formation using endoscopic intraoperative biopsies [1,2]. Although rats and mice are the most commonly used animal models in kidney stone formation research, dogs, pigs, and Drosophila have also been used.

In studies using animal models, stone rat models bred with oxalic acid precursors have been used frequently since the 1980s [3]. Conversely, a stone mouse model, which had previously been considered difficult to create, was reported for the first time in 2007 [4]. Crystal deposition was observed in stone rat models after hyperoxaluria was induced by exogenously administering sodium oxalate, glycolic acid, EG, and hydroxy-L-proline, among others [5–10]. In particular, crystal deposition is enhanced by increasing the urinary calcium concentration, and animal models receiving calcium chloride and vitamin D have been widely used for this purpose [11–13]. With the establishment of such stone animal models, the presence or absence of crystal deposition is primarily important, and many studies at the genetic level, including transcriptome analysis, have been performed [14–16]. Recently, urinary tract stone studies have investigated the involvement of oxidative stress and autophagy using stone model animals [17,18]. However, no study has actually explored early crystal development histologically or analyzed its cause and site of occurrence in detail, which is necessary.

The pathophysiology of Randall’s plaque formation has been studied from various perspectives [15]; however, few studies have investigated renal tissue and microenvironmental changes, such as tubular dilation occurring prior to plaque formation. Although previous reports have identified tubular dilation in the renal tissues of patients with kidney stones, the causal relationship between these changes and stone formation remains unclear.

A histological approach is needed to determine which sites in the renal tissue are involved in stone formation. We aimed to identify the site of crystal formation and the early histological changes associated with crystal formation, as well as to identify new factors involved in crystal formation by identifying the genes that are specifically upregulated in the tissue and that participate in stone formation.

## Methods

### Animals

All animal experiments were performed in accordance with the Guidelines for the Care and Use of Laboratory Animals and approved by the Experimental Animal Committee of Kanazawa Medical University (Permission number #2020−33). Ten-week-old male Wistar rats (SLC, Shizuoka, Japan) were housed in a room at constant temperature, humidity, and photoperiod (12 h light/dark) and fed Labo MR Stock (Nosan Corp., Yokohama, Japan) ad libitum.

### Preparation of the stone formation model rats

**Fig 1:**
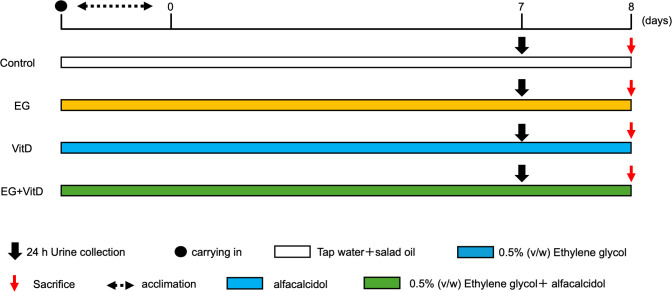
Experimental protocol.

Crystals were induced by oral administration of EG and alphacalcidol, a vitamin D3 analog, following the method of Okada et al [11]. Moreover, crystal formation was evaluated after 7 days of short-term rearing to avoid tissue failure due to stone formation. A scheme of the experimental setup is shown in Fig 1. Thirty-two 10-week-old normal male Wistar rats were divided into four groups: control, ethylene glycol (EG), vitamin D (VitD), and ethylene glycol + vitamin D (EG + VitD). Random numbers generated using the RAND function in Excel were used for group allocation. Based on the generated random numbers, each animal was randomly assigned to each group. After acclimatization for 2 days, the EG group was fed a 0.5% (v/w) ethylene glycol (Wako Pure Chemical Industries, Osaka, Japan) solution in tap water ad libitum, and the VitD group was fed 1.5 µg of alfacalcidol (Chugai Pharmaceutical, Tokyo, Japan; 0.5 µg/ml), which induces hypercalciuria, via a gastric tube. The VitD group was given 1 ml of alfacalcidol (0.5 µg/ml) every other day. The EG + VitD group received alfacalcidol every other day and 0.5% ethylene glycol solution ad libitum. The control group received 1 ml of salad oil instead of alfacalcidol and tap water (1 ppm chloride) instead of EG ad libitum for 6 days. On the seventh day, each rat was transferred to a metabolic cage, and 24 h later, the drinking volume was measured and urine was collected. Euthanasia was performed through carbon dioxide inhalation. Anesthesia was performed by intraperitoneally administering 10 mL/kg body weight of anesthetics (medetomidine, 0.15 mg/kg; midazolam, 2 mg/kg; and butorphanol, 2.5 mg/kg) in rats.

Thirty-two 10-week-old normal male Wistar rats were divided into four groups and acclimatized for two days after delivery. The rats were divided into control, ethylene glycol (EG), vitamin D (VitD), and ethylene glycol + vitamin D (EG + VitD) groups. Ethylene glycol (EG) in tap water (0.5%) was provided ad libitum. Active vitamin D (0.5 µg) was administered every other day as an alfacalcidol solution using a gastric tube. The control group received 1 ml of salad oil instead of alfacalcidol. Tap water (1 ppm of chloride concentration) was provided ad libitum in place of EG for 6 days; on day 7, each rat was transferred to a metabolic cage, and 24 h later, the drinking volume was measured, urine was collected, blood was drawn, and the rats were euthanized for sampling.

### Measurement of the urinary and serum variables

Urine was collected in a urine trap to determine the urine volume, and the urine calcium, magnesium, sodium, potassium, chloride, phosphorus, uric acid, and oxalic acid levels were measured. The serum Cr, blood urea nitrogen (BUN), calcium, sodium, potassium, chloride, uric acid, intact parathyroid hormone (PTH), magnesium, and phosphorus levels were measured using standard protocols provided by SRL (Tokyo, Japan). The urinary oxalate levels were measured using a FOM-110A (Meiden Hokuto Corp., Tokyo, Japan). The testing methods for each test item are as follows.

Urea nitrogen: Urease with GLDH, Creatinine: enzymatic method, Uric acid: Uricase,

Urinary oxalic acid: Capillary electrophoresis (CE), Na (sodium): Ion select elect DIL,

Cl (Chloride): Ion select elect DIL, K (potassium): Ion select elect DIL,

Ca (calcium): Arsenazo III DYE, P (inorganic phosphorus): Phosphomolybdate UV,

Mg (magnesium): Color-dye-xylidyl blue, Urea nitrogen (animal): Enzymatic method,

Creatinine (animals): Enzymatic method, Ca (animal): OCPC method, P (animal): Enzymatic method,

Na (animals): Enzymatic method, K (animal): Enzymatic method, Cl (animal): Enzymatic method.

### Kidney sampling and serial sectioning

All the rats were opened promptly after euthanasia. After left kidney removal, the whole rat body was deblooded in 0.9% saline and perfusion-fixed with 4% paraformaldehyde. The excised left kidney was divided into transverse half sections so that the cortex and medulla could be distinguished and further divided into cortex and medulla tissues (S1 Fig); the remainder was frozen. The right kidney was immersed and fixed in 4% paraformaldehyde. The fixed right kidney was divided in half and was embedded in paraffin for all groups. Four kidneys from each group were selected for tissue analysis, and each paraffin block was sliced into 5-µm slices using a microtome to prepare serial sections.

### Histochemical staining

The sections were double-stained with aquaporin1 and calbindin to stain the proximal and distal tubules, respectively. For the former staining, the primary antibody used was an anti-aquaporin 1 antibody [1/22] (Abcam PLC, Cambridge, UK; 1:750), and for the latter, an anti-calbindin antibody [EP3478] (Abcam; 1:1000) was used. The secondary antibodies used were Histofine Simple Stain Rat MAX-PO(M), Histofine Simple Stain Rat MAX-PO(R), Histofine Simple Stain AP(R), and Histofine Simple Stain AP(M) (all Nichirei Biosciences Inc., Tokyo, Japan). The substrates were also stained using a Vina Green Chromogen Kit (Funakoshi Co., Ltd., Tokyo, Japan), New Fuchsin Substrate Kit (Nichirei), and a Simple Stain DAB solution (Nichirei). Calcium oxalate crystals were stained using Pizzolato stain using a Calcium Stain Kit (Modified Von Kossa, Scy Tek Laboratories, Inc., West Logan, UT, USA) and observed using a microscope. Hematoxylin and eosin (HE) staining was performed using New Hematoxylin Type R and New Eosin Type R stains (Muto Pure Chemicals Co., Ltd., Tokyo, Japan).

### Identification of the crystal formation sites and tubular segments

The structure of rat and human kidneys is different. For this reason, McFarlane classified rat kidneys into zones, reported three types of nephrons, and illustrated the predominant nephron sites in each zone [19]. Here, we followed this report, divided the rat kidney into three types of nephrons in the transverse half section, and identified the respective regions in the kidney.

First, two consecutive sections that were considered observable without tissue contusion within an interval of 50 slices in the EG + VitD group were selected. The H&E-stained sections were classified as: Zone I, outer cortex; zone II, inner cortex; zone III, outer medulla; and zone IV, inner medulla.

H&E-stained, zone-classified, and Pizzolato-stained mapped sections were superimposed using Photoshop (http://www.adobe.com/products/photoshop), and the number of crystals in each zone was counted and documented.

The crystal-forming tubular segments were investigated by staining serial sections from the EG + VitD group. The crystals were washed out, and the contrast was insufficient, making observation difficult. Therefore, comparative images of serial sections were used for observation. The proximal tubules, distal tubules, and calcium oxalate crystals were stained with aquaporin1, calbindin, and Pizzolato stains, respectively, to identify each tubule segment and crystal. Sections double-stained with aquaporin1 and calbindin and Pizzolato-stained serial sections 50 slices apart were randomly selected. The crystals were analyzed to determine which tubules were present in a randomly selected 1 mm^2^ region where crystals were present. Similarly, calbindin-, aquaporin1-, Pizzolato-, and HE-stained images from six sets of serial sections selected at 50-slice intervals were compared to identify the tubular segments where crystals were formed.

### Measurement and comparison of the lumen area

Kidney tissue sections from eight rats in each group were double-stained with aquaporin1 and calbindin, and the center of the 9 mm^2^ area was used as the measurement sample. A random and systematic sampling method [20] was then used to determine the total area S, intraluminal area S1, and cell area S-S_1_ of the aquaporin1-positive tubules within the randomly selected measurement area. Photoshop was used to measure the area, and the tubular intraluminal cell density = (S-S_1_)/S was determined to compare the degree of dilation of the proximal tubular lumen.

### RNA extraction and cDNA generation

The collected rat kidney tissue was homogenized using a BHA-6 bead milling machine (As One Corp., Osaka, Japan), and the total RNA was extracted using an RNeasy Mini kit (QIAGEN N.V., Venio, Netherlands) [21]. Agilent RNA 6000 Nano Assay (Agilent Technologies, CA, USA) was used for quality check, and the total RNA with a RIN ≥ 7.0 was used for further analysis.

### Transcriptome analysis using DNA microarray

cDNA samples were prepared using a GeneChip WT PLUS Reagent Kit 90228, GeneChip Hybridization, Wash and Stain Kit, and GeneChip Rat Gene 2.0 ST Array (Thermo Fisher Scientific Inc., MA, USA). Gene networks were evaluated using GeneSpring software and IPA (Ingenuity Systems, QIAGEN). Two microarray analyses were performed in the cortex and medulla, and the genes with a fold-change ≥ 2.0 were selected as variable genes, and the genes that were variable in both analyses were designated as genes with variable expression.

### Real-time PCR analysis

Reverse transcription was performed using a ReverTra Ace qPCR Master Mix with qDNA Remover (FSQ-301, Toyobo) to synthesize cDNA from the extracted total RNA. TaqMan® Fast Advanced Master Mix for qPCR (4444557), TaqMan® Gene Expression Assays (Applied Biosystems, Thermo Fisher), and cDNA were mixed and analyzed using a QuantStudio 12 K Flex Real-Time PCR System (Life Technologies, Thermo Fisher). In quantitative PCR, the following genes were targeted: FAM-MGB 18S rRNA (Hs99999901-s1), *Slc23a3* (Rn01400103-m1), *Bsnd* (Rn00594503-m1), *Aqp2* (Rn00563755-m1), *Aqp3* (Rn00581754-m1), *Cldn16* (Rn00590884-m1), *Fga* (Rn01462588-m1), *Slc7a9* (Rn00588400-m1), *Slc4a1* (Rn00561909-m1), *Trpv5* (Rn00587268-m1), and *Slc7a7* (Rn00580189-m1).

### Analysis of tissue FGA expression using immunostaining

Frozen sections (5–6-µm-thick) were used for immunostaining. A 100-fold dilution of fibrinogen alpha chain recombinant rabbit monoclonal antibody (ARC2227, Invitrogen, Thermo Fisher Scientific) was used as the primary antibody against FGA. The secondary antibody used for Histofine simple staining of rat tissue was MAX-PO (MULTI) 414191 (Nichirei). The specimens were stained with DAB and observed using an optical microscope.

To account for the distribution heterogeneity, all fields of the tissue sections were scanned at low magnification (original magnification: 40×). The number of cells stained and the staining pattern were recorded; stromal and lymphoid cells were excluded. The immunoreactivity of each section was quantitatively assessed by evaluating the percentage of positive cells (especially proximal tubular epithelial cells) relative to that of tubular epithelial cells. All samples were evaluated by an independent observer (pathologist in our team: J.H.) using a blind protocol design. The results showed that the inter-observer agreement, as measured by the interclass correlation coefficient, was greater than 95%. In a few (< 5%) cases of disagreement, a consensus score was attributed by a second pathologist (S.Y.).

### Investigation of the molecular binding sites of FGA by OxaBIND

Access the OxaBIND database (https://www.stonemod.org/oxabind.php), FGA protein codes obtained from Uniprot (https://www.uniprot.org) (P02671 - FIBA_HUMAN) was entered and investigated. The date and time of access (2023/8/7).

### Statistical analysis

A sample size of eight animals per group was used in this study. The choice of sample size was based on resource constraints and animal welfare considerations. A preliminary power analysis indicated that a sample size of approximately 129 animals per group would be required to detect a moderate effect size (Cohen’s f = 0.25), but due to practical constraints a sample size of 8 animals per group was used. The power of this study is therefore estimated to be approximately 50%. All data are expressed as the mean ± SD. One-way analysis of variance was used for comparisons among the four groups, and the Student’s T-test was used for comparisons between two groups. To check for normality and homocedasticity, respectively, the Kolmogorov-Smirnov and Bartlett tests were used. All analyses were performed using EZR statistical software (version 1.63, Saitama Medical Center, Jichi Medical University, Saitama, Japan) and GraphPad Prism version 10.3.0 for Windows (GraphPad Software, Boston, Massachusetts USA, www.graphpad.com).

## Results

### Urinary and blood findings

**Fig 2:**
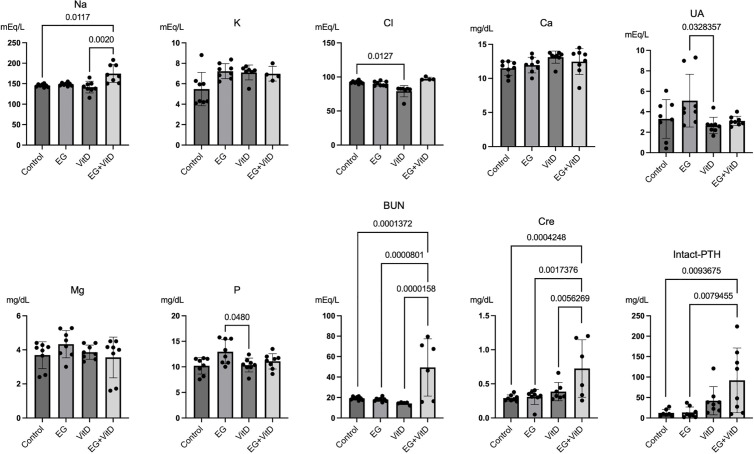
Results of the biochemical analysis of the blood samples. Four groups of rats (N = 8) (control, EG, VitD, and EG + VitD groups) were kept for 7 days and sampled after euthanasia. EG, ethylene glycol group; VitD: Vitamin D group; EG + VitD, ethylene glycol + Vitamin D group. Multiple comparisons were made using one-way analysis of variance (ANOVA), and the results are presented as the mean ± SD. *P < 0.05.

**Fig 3:**
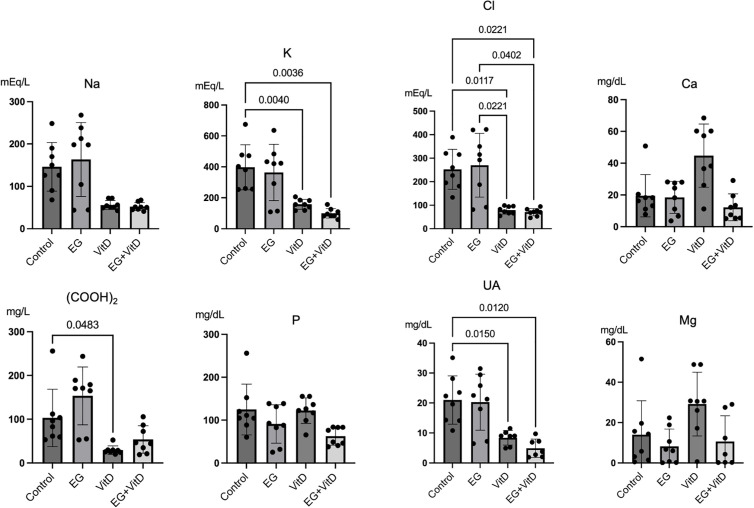
Results of the biochemical tests of rat urine. Urine was sampled 24 h after the 6-day-long treatment was finished. The results are presented as the mean ± SD. Multiple comparisons were performed using one-way analysis of variance (ANOVA). *P < 0.05.

First, to confirm the changes in the levels of urinary stone formation-related components in urine and blood, we analyzed the 24-h urine stores collected after rearing and the blood samples collected before euthanasia. Blood test results showed that the levels of BUN and creatinine, which are renal function parameters, increased in the EG + VitD group (Fig 2). Moreover, the intact PTH level was significantly higher in the EG + VitD group than in the control group. The blood calcium levels did not significantly differ among the groups. In addition to sodium, potassium, and chloride, uric acid excretion decreased in the VitD and EG + VitD groups (Fig 3). Urinary calcium was higher in the VitD group than in the control group, and oxalic acid excretion was lower in the VitD and EG + VitD groups than in the control group. These data confirm that the change in chloride concentration in urine compared to that in the control group was higher than that observed in blood. This suggests that crystal formation is largely related to events occurring after the transglomerular passage.

### Site of crystal development and tubular dilation in the EG + VitD group

Pizzolato staining of the kidney sections from each of the four groups revealed that crystals were only present in the EG + VitD group.

**Fig 4:**
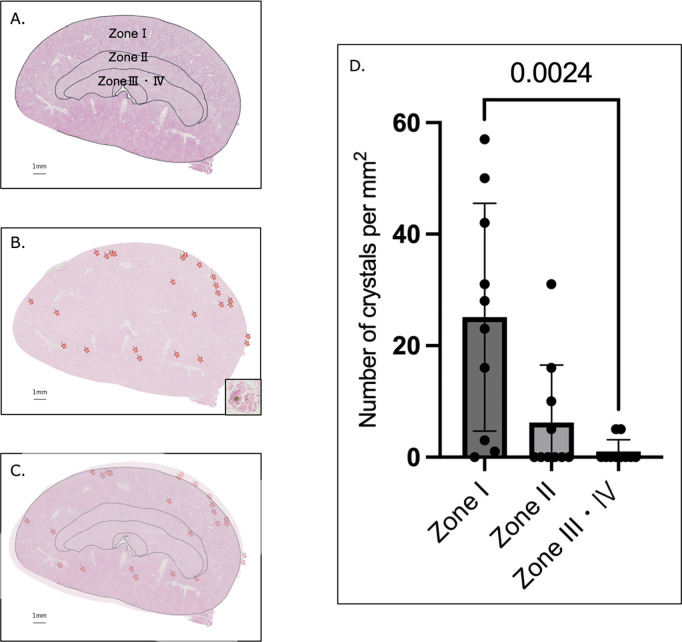
The initial crystal formed by EG + VitD administration is centered in the lateral cortex. Serial sections (5-µm-thick) were prepared from the EG + VitD group renal specimens that showed crystal formation, and two sections at a 50-slice interval were selected. Hematoxylin and eosin (HE) and Pizzolato staining were performed for section n and n + 1 in this order. Representative images are shown in (a)–(c). (a) Section n, which was selected for tissue observation, was subjected to HE staining and each of its tissue regions was identified and separated (Zone I: outer cortex; zone II: inner cortex; zone III: outer medulla; and zone IV: inner medulla). (b) Section n + 1 was stained with Pizzolato stain, and all the observed crystal locations are marked. (c) Overlay of images (a) and (b). (d) The number of crystals aggregated in each zone is shown for comparison. Renal tissue sections were imported into Nanozoomer 2.0 – HT Slide Scanner by Hamamatsu (https://www.hamamatsu.com/jp/ja/product/life-science-and-medical-systems/digital) and observed using NDP.view2 (image viewing software U12388-01). Values are presented as the mean ± SD. *P* < 0.05. The P-values were calculated using one-way analysis of variance (ANOVA) and the Tukey–Kramer method using EZR (version 1.63) and GraphPad Prism software (version 10.3.0).

To determine which region of the kidney the early crystals begin to develop in, Pizzolato staining was performed on the renal sections of the EG + VitD group, in which crystals had formed, to visualize crystal formation (representative staining examples are shown in Fig 4A–C). Each renal section region was classified as described in the Methods. The number of crystals formed in each region was counted. This number was the highest in zone I, the lateral cortex (Fig 4D), followed by that in the medial cortex (zone II) and medulla (Zones III + IV). This indicates that the early crystals may have originated from the tubular segments predominantly distributed in zone I.

**Fig 5:**
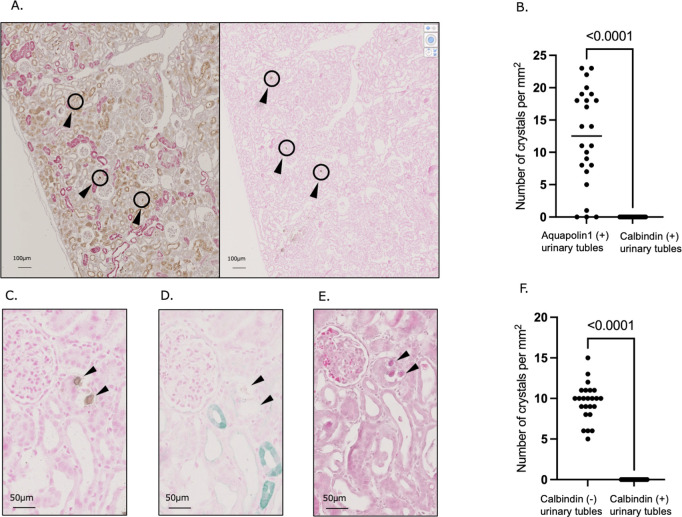
The initial crystal arises from the proximal tubules. From the serial sections (5-µm-thin) of the rats in the EG + VitD group (N = 4), two sections at a 50-slice interval were selected. (a) From the plot of crystals that could be observed by staining section n with Pizzolato stain, the locations corresponding to a 1 mm^2^ area were randomly selected (S2 Fig). Section n + 1 was double-stained with aquaporin1 and calbindin (to stain the proximal and distal tubular cells, respectively) to determine the type of tubular segment to which the crystal corresponds within a randomly selected region from section n. (b) The number of crystals present in the aquaporin1- and calbindin-positive tubules is indicated. (c) The crystal formation sites were randomly selected from the Pizzolato-stained tissue section n. Arrows indicate the crystals formed in the tubules. (d) The tissue section n in which crystals were identified was stained with calbindin. Arrows indicate the sites where crystals were found after Pizzolato staining. (e) HE staining of the tissue section n + 1 and comparison of the same areas with those in (c) and (d). Arrows indicate the locations where crystals were found. (f) In the area compared between sections n and n + 1, the presence or absence of crystals in the calbindin-positive and -non-positive tubules was registered by comparing images such as those in (c), (d), and (e). In (b) and (f), the P-values were calculated using Student’s t-test using GraphPad Prism software (version 10.3.0) after establishing the null hypothesis. The values are presented as the mean ± SD. *P < 0.05.

Multiple staining and serial section analyses were performed to determine in which tubular segments more crystals were formed initially (see S2 Fig for an example of the random selection method used for the renal sections). These results confirm that many crystals were in the proximal tubules within a randomly selected area (Fig 5A, B). Furthermore, the Pizzolato-stained sections were compared with the consecutive sections double-stained with calbindin to identify the location of crystal formation and showed that the crystals were located in a calbindin-negative tubule within the selected region (Fig 5C–F). Therefore, the initial crystals in the early phase are likely to have mainly formed in the proximal tubules.

**Fig 6:**
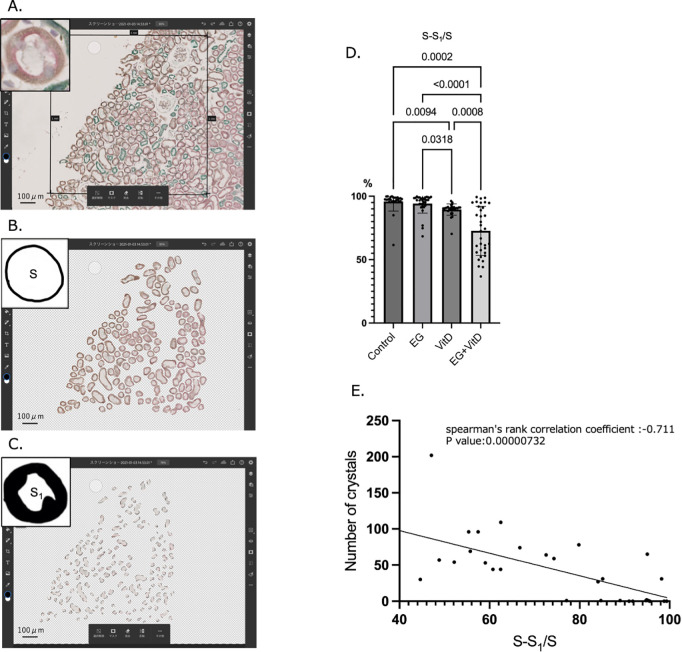
The proximal tubules in the EG + VitD group in which crystals were formed are significantly more dilated than those in the other groups in which crystals were not formed. (a) The renal tissue sections from each group (N = 8) were double-stained with aquaporin1 and calbindin, and the center of the 9 mm^2^ area was used as the measurement sample (S3 Fig). A randomly selected measurement sample area (1 mm^2^) was captured in an image using Photoshop. (b) Only the aquaporin1-positive tubules (proximal tubules) within the 1 mm^2^ area were cropped, and the total area was calculated. The upper left shading represents the area S, which includes proximal tubular cells and the tubular lumen. (c) From the cropped proximal tubule image (S), only the tubular lumen area (S1) was cropped, and the total area was calculated. (d) The intraluminal density of tubular component cells was compared using the formula (S-S_1_)/S. The P-values were calculated using one-way analysis of variance (ANOVA) and the Tukey–Kramer method using EZR (version 1.63) and GraphPad Prism software (version 10.3.0). The values are presented as the mean ± SD. *P < 0.05. (e) The correlation between the number of crystals formed and proximal tubular dilation was investigated in the EG + VitD group rats, in which crystal formation was confirmed. Spearman’s rank correlation coefficient was used for statistical analysis with a significance difference of *P* < 0.05.

Renal tissue specimens from each group showed prominent tubular dilation in the tubules of the EG + VitD group, in which stone formation was observed. Therefore, double staining was performed to identify the segments corresponding to the dilated tubules (Fig 6A–C). A representative image of the random selection method used for choosing the kidney study area is shown in S3 Fig.

The proximal tubules in the EG + VitD group were more dilated than those in the control group (Fig 6D). Furthermore, the higher the number of crystals formed in the EG + VitD group, the lower the intraluminal cell density (Fig 6E).

### Gene expression analysis using microarray and quantitative RT-PCR

The above immunohistochemical analysis suggests that the event involved in crystal formation occurred in the proximal tubules. To investigate the molecular changes occurring in the proximal tubules and the novel gene target, we divided the kidneys of each group of rats into the cortex and medulla, extracted the total RNA, and performed DNA microarray analysis.

**Fig 7:**
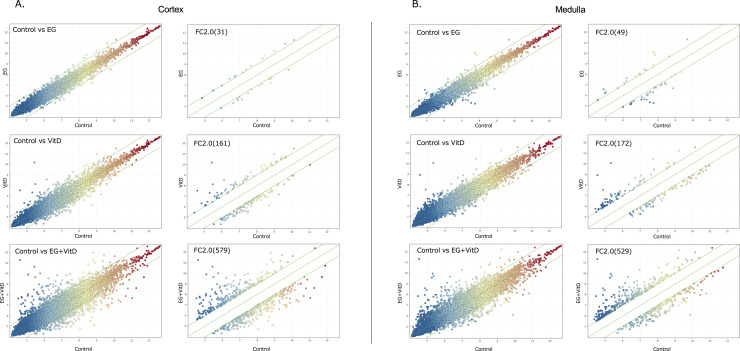
DNA microarray analysis results. Scatter plots were used to evaluate the mRNA expression variation among the three groups of rats and the control group. (a) Scatter plots of the cortex in the control group rats and in those of each of the three other groups. (b) Scatter plots of the medullae in the control group rats and in those in each of the other three groups. The values on the x- and y-axes of the scatter plots are the normalized (log2 transformed) values for each sample. The green line represents the fold-change (the default fold-change value is 2.0). The mRNAs above the upper green line and below the lower green line have a 2.0-fold-change in mRNA expression among the three groups. The results of the microarray analysis of the crystal formation group (EG + VitD group) are posted as supplementary data. (S4 Fig.).

**Fig 8:**
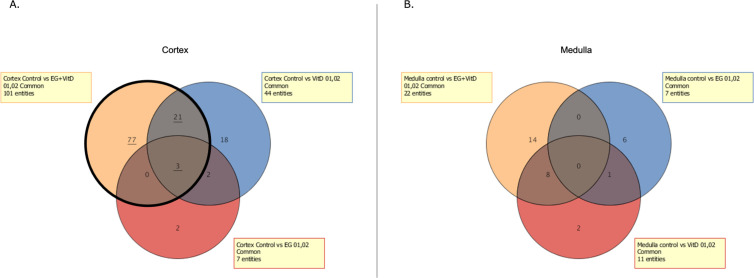
Venn diagram showing the number of variable genes in the EG, VitD, and EG + VitD groups relative to the control group. Each variable gene was selected from two independent microarrays (fold-change = 2.0). (a) Venn diagram showing the variable genes in the cortex. Bold lines indicate the number of genes in the EG + VitD group, which were the most abundant among the extracted variable genes. (b) Venn diagram showing the variable genes in the medulla.

**Fig 9:**
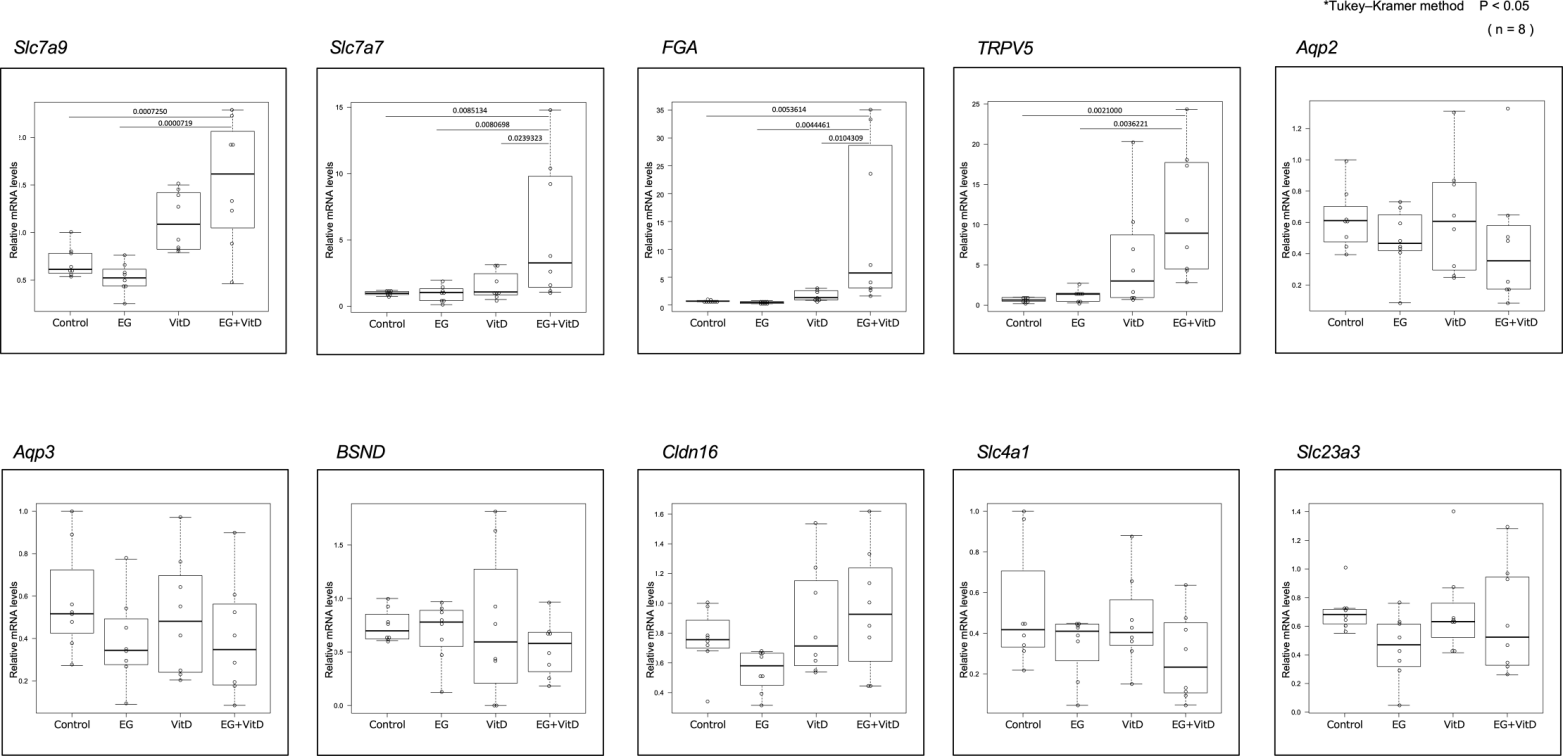
Gene expression analysis of the rat kidney using real-time PCR (qPCR). The gene expression levels were normalized to those of the housekeeping gene (18S rRNA) and the data are expressed as absolute quantification values using the standard curve method. Statistical differences between groups were calculated using the Tukey-Kramer method and one-way analysis of variance (ANOVA). Values are presented as the mean ± SD. *P < 0.05 (N = 8). *Slc7a9*: solute carrier family 7 member 9; *Slc7a7*: solute carrier family 7 member 7; *FGA*: fibrinogen alpha chain; *TRPV5*: transient receptor potential cation channel subfamily V member 5; *Aqp2*: aquaporin 2; *Aqp3*: aquaporin 3 (gill blood group); *BSND*: barttin CLCNK type accessory subunit beta; *Cldn16*: claudin 16; *Slc4a1*: solute carrier family 4 member 1 (Diego blood group); *Slc23a3*: solute carrier family 23 member 3.

**Fig 10:**
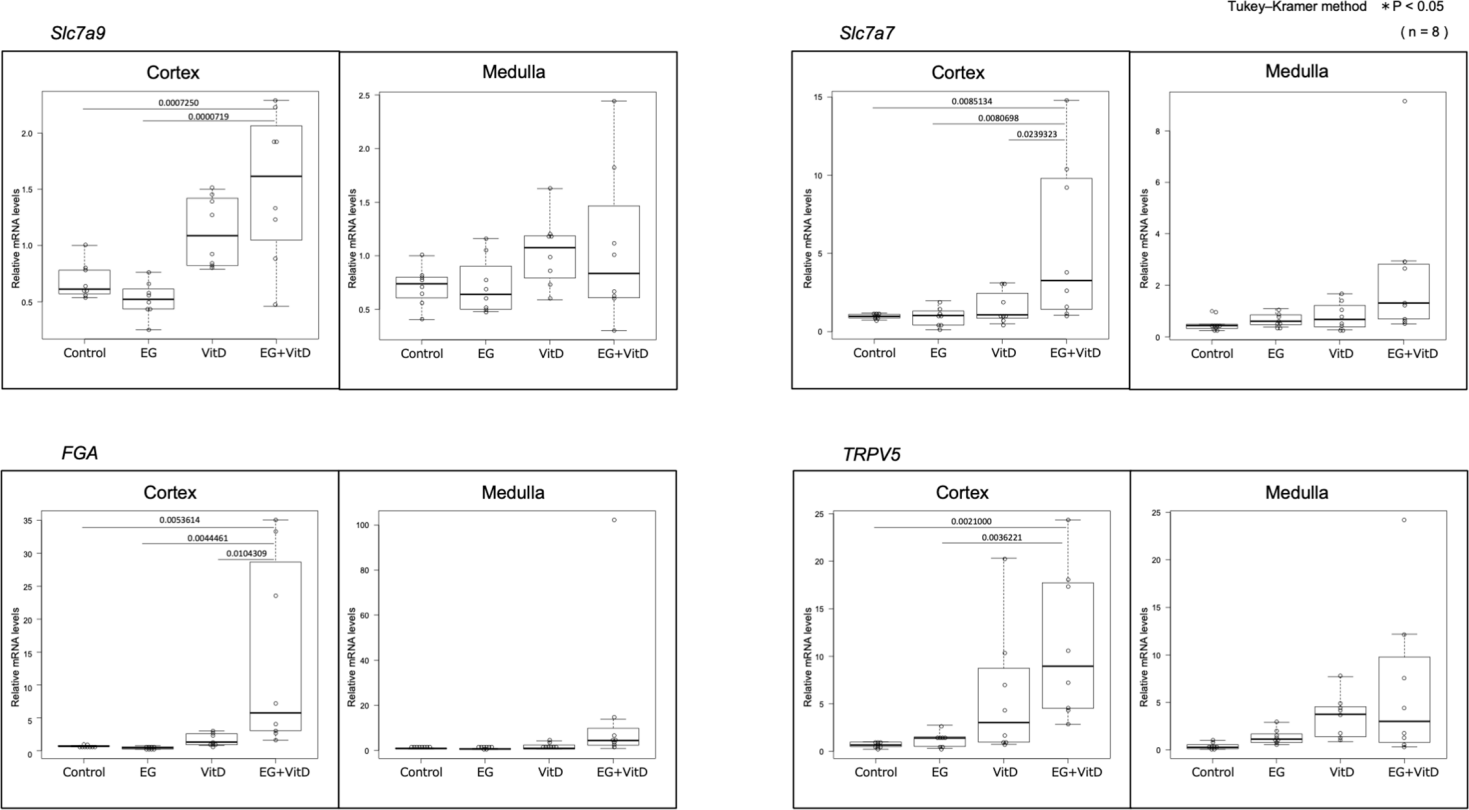
Comparison of the gene expression in the cortex and medulla for the four genes with high gene expression levels in the EG + VitD group. Cortical and medullary specimens were frozen immediately after debridement and blood removal and were divided at the border between the cortex and medulla, which could be seen with the naked eye under translucent light (S1 Fig). The gene expression levels were normalized against those of a housekeeping gene (18S rRNA), and data are expressed as absolute quantification using the standard curve method. Statistical differences between groups were calculated using the Tukey-Kramer method and one-way analysis of variance (ANOVA). The values are presented as the mean ± SD; *P < 0.05 (N = 8).

In both the cortex and medulla, the number of genes with differential expression in the EG + VitD, VitD, and EG groups increased, in that order, compared to that in the control group (Fig 7A,B). In two independent microarray analyses, the genes that were commonly altered with a fold-change of 2.0 or higher were extracted from the genes that were altered in the EG + VitD group. As a result, 101 and 22 genes were extracted from the cortex (Fig 8A) and medulla (Fig 8B), respectively, compared to the control group. Gene Ontology analysis was performed using IPA to examine the mRNA expression profiles. The results showed that the Renal and Urological Disease categories were among the top three. Based on the functional evaluation results obtained using IPA and the literature, 10 urolithiasis-related genes were selected. The expression levels of these genes were quantified using RT-PCR (Fig 9). Quantitative RT-PCR (N = 8 per group) of the 10 extracted genes was performed, and significant *Slc7a9, Slc7a7,* fibrinogen alfa chain (FGA), *and TRPV5* gene expression changes compared to those of the control group were observed. The expression levels of these four genes in the medulla and cortex were compared, and those in the cortex were significantly upregulated for all the groups (Fig 10). Among these genes, we focused on *FGA*, which has not been associated with urinary tract stone disease, and analyzed the FGA protein expression.

### FGA immunostaining

**Fig 11:**
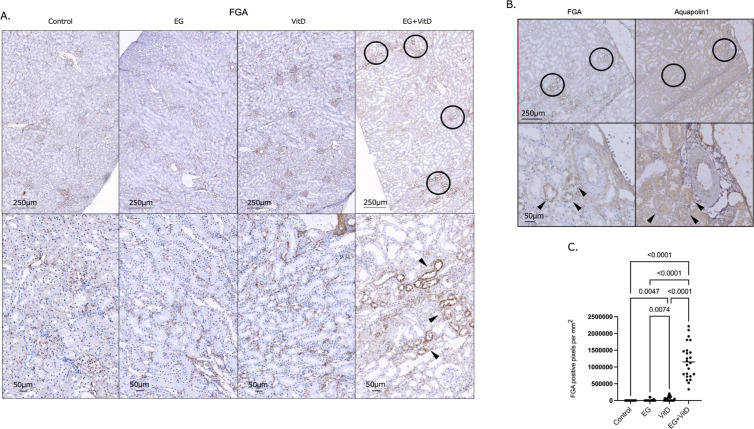
Quantification of FGA expression using immunohistochemistry. Immunohistochemically stained renal tissue sections were imported into Nanozoomer for observation using NDP.view2. (a) FGA immunostaining was performed for each of the four groups (N = 8), and protein expression was compared among groups. The magnification shown on the left side of the image is the magnification of the digital microscope and was observed on a 34-inch monitor. Circles indicate the FGA-positive tubules observed at low magnification. Arrows indicate the FGA-positive tubules, which were identified at a higher magnification. (b) Serial sections (n to n + 1) were used to investigate the type of tubular segments of the FGA-positive tubules, and the regions that matched those identified as FGA-positive tubules on section n were extracted and verified on section n + 1 (N = 8). Representative images are shown. Circles indicate the FGA-positive tubules in section n, identified at weak magnification, and the corresponding tubular sites in section n + 1. Arrows indicate the FGA-positive tubules of section n and the corresponding tubules of section n + 1 observed at high magnification. (c) The total area of FGA-positive tubules was compared among the four groups. The measurements were performed using Photoshop, quantified, and compared in pixel units for verification. Statistical differences between groups were calculated using the Tukey-Kramer method and one-way ANOVA. The values are presented as the mean ± SD. *P < 0.05 (N = 8).

To determine the FGA protein expression and identify the tubular segments expressing FGA, FGA immunostaining was performed for the four groups (N = 8), and the stained tubular areas were quantified and compared using Photoshop software. For each group, three points in one section were randomly selected for analysis, and a tubule-specific immunostaining image of FGA was only observed in the cortical-predominant and EG + VitD groups (Fig 11A).

To clarify which tubule segments were stained, serial sections were stained with aquaporin1, and FGA expression was consistent with the aquaporin1-positive tubules (proximal tubules) (Fig 11B). The tubule area immunostained for FGA was compared among the four groups, and FGA was predominantly expressed in the EG + VitD group (Fig 11C). These results indicate that FGA was predominantly expressed in the proximal tubules of the EG + VitD group.

### Ca^2+^ and oxalate-binding capacity of FGA

**Fig 12:**
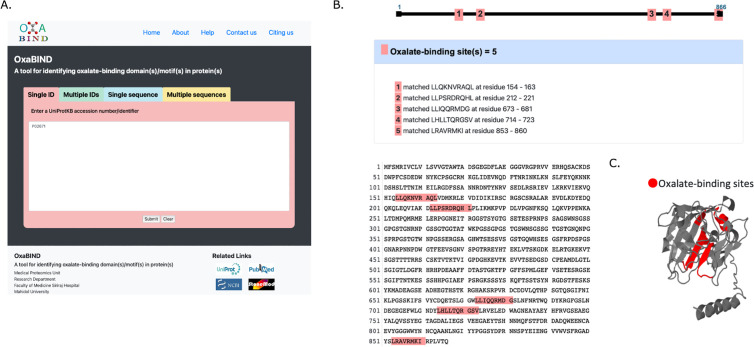
Molecular analysis of the FGA.

As previous results have shown that FGA has significant gene and protein expression in the proximal tubules, the possibility that FGA is directly involved in oxalate crystal nucleation was investigated. First, the FGA-binding sites for Ca2^+^ were investigated from Uniprot, and it was confirmed that there are four sites that can bind Ca^2+^. Similarly, for oxalic acid, the FGA protein code from the OxaBIND database was used to examine the oxalic acid molecular structure and possible binding sites with FGA, and five sequences were predicted to encode oxalic binding sites in FGA protein. (Fig 12A–C)

The single ID of FGA from the OxaBIND (https://www.stonemod.org/oxabind.php) website was entered, and “Submit” was clicked to start the analysis. (a) The site where oxalic acid can bind to the FGA protein was found and selected, and the possible binding site was displayed in red on the protein code. (b) 3D conversion was performed, and the oxalic acid-binding site of the FGA protein was displayed and confirmed in 3D.

## Discussion

Here, calcium oxalate crystal deposition in the proximal tubules was observed in the EG + VitD group during the early phase. While stone formation has been reported in Okada’s stone rat models within four weeks after dosing, here, crystal formation was observed within a short period of seven days. Moreover, in previous urinary tract stone studies, crystals were often observed in the cortical-medullary border region; [6,22–24] however, here, crystal deposition in the early phase was different. These differences may depend on the time course, timing, and observation method used after reagent administration, as well as the onset of the disease, which creates a crystal formation-inducive environment. Khan et al. concluded that the initial crystals form in the proximal tubular lumen of the renal cortex and that the crystal size, number, and distribution depend on the amount of reagent injected and the time interval post-injection [5]. Oxalic acid is excreted in urine from the proximal tubules [25,26], and here, excessive filtered oxalic acid excretion was observed in the EG-treated group. Conversely, 55% of the Ca^2+^ filtered from the glomerulus is reabsorbed in the PCT (proximal convoluted tubule), 10% is reabsorbed in the PST (proximal straight tubule), 20% in the TAL (thick ascending limb of Henle’s loop), 10% in the DCT (distal convoluted tubule) and CNT (connecting tubule), and the remaining 2% is reabsorbed in the CCD (cortical collecting duct), with 1–3% excreted in the urine [27]. Moreover, the resorption mechanism involves the tight junction proteins claudin2 [28,29], and although no significant gene expression variation was observed here (S5 Fig), crystal-forming tubule segments were shown to be involved in stone formation. While oxalic acid and calcium concentration are important factors in the formation of crystals, we must not forget the secondary predisposing factors that affect the environment in which crystals form. For example, it is possible that oxalate or calcium acted on citric acid or magnesium concentrations to create an environment in which calcium oxalate crystals were likely to form in the proximal tubule [30]. As reported in previous studies [31–34], Mg^2+^ is listed as a suppressor of calcium oxalate stones. Few studies have reported events wherein urinary magnesium is related to citrate concentrations. However, the detailed molecular mechanism remains unclear. Various other factors may be involved in early crystal formation.

Although tubular dilation occurs in stone-forming renal tissues, a causal relationship between stone formation and tubular dilation has not been clearly established. EG may inhibit mitochondrial respiration [35], increase LDH activity [36], activate caspase-3, and cause renal tubular dilation resulting from the apoptosis of renal epithelial cells [37,38]. Furthermore, renal tubular epithelial cell damage due to oxidative stress induction is associated with mitochondrial instability, leading to histological changes [39]. Tubular dilation is used as a defense mechanism that involves the activation of PKD-related signaling pathways throughout crystal formation [40], and its histological pathophysiology is complex and multifactorial. *Slc7a7*, whose expression was significantly upregulated here, is associated with proximal tubular brush border loss and tubular dilation; however, their relevance to stone formation and the corresponding histological changes are unclear [41]. As for the report in this knockout model, we speculate that gene network feedback(s) may be occurring, since the expression is upregulated in the opposite direction. Mutations in the SLC7A9 (also SLC3A1) gene are a common cause of cystinuria and lead to the formation of cystine stones. This gene encodes a subunit of the amino acid transport system, and its mutation leads to cystine accumulation [42–46]. Rare missense mutations in the TRPV5 gene have been reported to be associated with recurrent kidney stones. However, since TRPV5 acts in Ca reabsorption, high expression of TRPV5 is generally reported to be associated with urinary tract stone prevention [47,48]. So far, no reports of FGA and Slc7a7 in UTI have been found, although Slc7a7 mutations have been implicated in lysinuric protein intolerance (LPI), which has been reported more frequently [49].

Here, we quantitatively demonstrated that the proximal tubule was the segment that underwent tubular dilation (Fig 6A–D), which correlates with the number of crystals formed (Fig 6E). The significant cortical expression of *FGA* and other stone formation-related genes (Fig 10) indicates that the proximal tubules are significantly involved in early crystal formation, and their protein expression suggests that FGA might be involved in early crystal formation (Fig 11A–C). Here, the flattening of the renal tubular epithelial cells was the basis for the resulting proximal tubular dilation. Epithelial cell flattening is largely related to the loss of polarity of the constructed cells [50,51]. The action of FGA on the integrins and microtubules involved in the cytoskeleton and tight junctions may affect cell polarity.

The crystal formation process requires a persistent supersaturation of the stone constituents, and the involvement of microscopic organic compounds is important for crystal nucleation [52]. Matrix proteins are constituents of calcium oxalate stones, and in the 1960s, the apparent presence of organic components in these stones was demonstrated [53]. In other words, FGA is an organic compound that may be an important factor in inducing crystal nucleation in the proximal tubules (Figs 9–11).

With the recent advances in analytical techniques, more than 1000 proteins in stones have been identified using proteomics, and 20 of the most frequently observed matrix proteins have been reported [54]. FGA is one of the most commonly reported matrix proteins and is a biomarker used for a noninvasive diagnosis of urinary tract stone disease [55]. However, few reports have specifically shown its involvement in calcium oxalate stone formation. Here, RT-PCR and immunostaining revealed that FGA was strongly expressed in the proximal tubular center of the EG + VitD group, suggesting that it may contribute to the salting-out effect of calcium oxalate crystals in the proximal tubules at an early stage of crystal nucleation. FGA is an alpha component of the glycoprotein fibrinogen, consisting of three pairs of nonidentical polypeptide chains, and glycoproteins promote calcium oxalate crystallization at high concentrations [56]. We used the FGA protein code from the OxaBIND database to examine the possible oxalic acid-binding sites in FGA according to the oxalic acid molecular structure. Five of these potential binding sites were identified in FGA (Fig 12) [57]. Furthermore, four calcium-binding sites were identified in FGA, suggesting that it may have affinity for both calcium and oxalic acid. Organic substances such as osteopontin (OPN), renal prothrombin fragment 1 (RPTF-1), and calgranulin A (Cal-A) exist in most CaOx crystals and are calcium-binding proteins that may influence CaOx crystal formation. These proteins have calcium-binding domains [58–60], and their adsorption and incorporation into CaOx crystals are influenced by the binding strength of the amino acid side chains and the protein-specific properties, which are conditioned by electrostatic charges [61,62]. Thus, the affinity of local proteins for positively-charged Ca^2+^ ions on the crystal surface leads to crystal nucleation and growth. Conversely, FGA has a direct oxalic acid-binding site and may be crucial in CaOx crystal growth. Further investigation of the protein distribution and physicochemical properties of FGA in oxalic acid crystals is necessary. Therefore, genetically modified rats are useful but only mouse models are available now [4]. We would like to proceed with the analysis from the creation of genetically modified rats in future studies. It is also possible that high molecular weight proteins such as FGA may interfere with endocytosis in the proximal tubules by coating calcium oxalate crystals [63]. For example, Tamm-Horsfall protein has been suggested to also inhibit crystal adhesion to renal cells [64] and the distribution and physicochemical properties of FGA in calcium oxalate crystals require further investigation.

FGA contains RGD (Arg-Gly-Asp) in its molecular structure, which has a strong affinity for integrin α5β1 [65], and the mechanical effects induced on integrin or integrin function-regulating proteins may cause cell polarity disorder, leading to histological changes such as tubular dilation. Further analysis is required to determine whether the expression of these genes and FGA is associated with histological changes such as proximal tubular dilation. The limitation of this study is that it was a relatively short-term rat model of stone formation, and the involvement of the FGA gene was concluded from in silico analysis inspired by the gene expression variation. However, unlike mouse and other model animals, FGA gene-modified rats are not available, making it difficult to provide direct proof. The main therapeutic strategy in this study will be to prevent stone formation. Without understanding the origin of early crystal formation, we will not be able to determine which pathological mechanism to stop in order to show recurrent effects. Although it is still only a hypothesis, if the FGA plays a role as a catalyst in the early stage of crystal formation (whether alone or in combination with other upregulated genes), we can expect a decrease in crystal formation during FGA inhibition.

However, whether FGA is abundant in the proximal tubules, whether it contributes to the crystal precipitation effect, and by what mechanism crystal formation takes place need to be clarified. Specifically, if FGA is involved, blocking FGA secretion, blocking the FGA binding site, or changing the FGA structure may be considered.

## Supporting information

S1 Fig Separation of the renal cortex and medulla. The photograph shows the left kidney, ligated and removed after euthanasia, and then split in half, showing a transverse half section. Each kidney was divided using a scalpel at the line separating the cortex and medulla under translucent light and then cryopreserved.
(TIFF)

S2 Fig Understanding the crystal growth region. Serial sections of the EG + VitD group in which crystals had formed were stained with Pizzolato stain, and all the crystals that could be identified were plotted. A 1 mm^2^ grid was then applied, and the crystal-forming tubule segments were counted within the randomly selected area of the grid.
(TIFF)

S3 Fig Double staining of renal tubules and randomised measuring area. EG + VitD kidney sections double-stained with aquaporin1 and calbindin were fitted to a 1 mm^2^ grid, and the area corresponding to the central 1 mm^2^ box was selected as the measurement site in the 9 mm^2^ box area.
(TIFF)

S4 Fig The results of the microarray analysis of the crystal formation group (EG + VitD group). The results of the microarray analysis of the crystal formation group (EG + VitD group) are published as supplementary data. In addition, all microarray data from this study are available under GSE269407 in NCBI-GEO.
(PPTX)

S5 Fig Results of real-time PCR for the Claudins gene group. Results of real-time PCR for Claudin genes (Claudin2, Claudin10, Claudin3, Claudin19) involved in the regulation of Ca2+ in the renal tubules. The gene expression levels were normalized to those of the housekeeping gene (18S rRNA) and the data are expressed as absolute quantification values using the standard curve method. Statistical differences between groups were calculated using the Tukey-Kramer method and one-way analysis of variance (ANOVA). Values are presented as the mean ± SD. *P < 0.05 (N = 8).
(TIFF)

S6 File The minimal data set. Tables showing the results of Figs 2–11 and S5.
(XLSX)
