# Supplementary figures and images for: CaOx crystal nuclei are formed in rat outer cortex proximal tubules by a potential fibrinogen-dependent mechanism
Source: PLoS One. 2025 Sep 9;20(9):e0328721. doi: 10.1371/journal.pone.0328721 (PMC12419635; doi:10.1371/journal.pone.0328721)

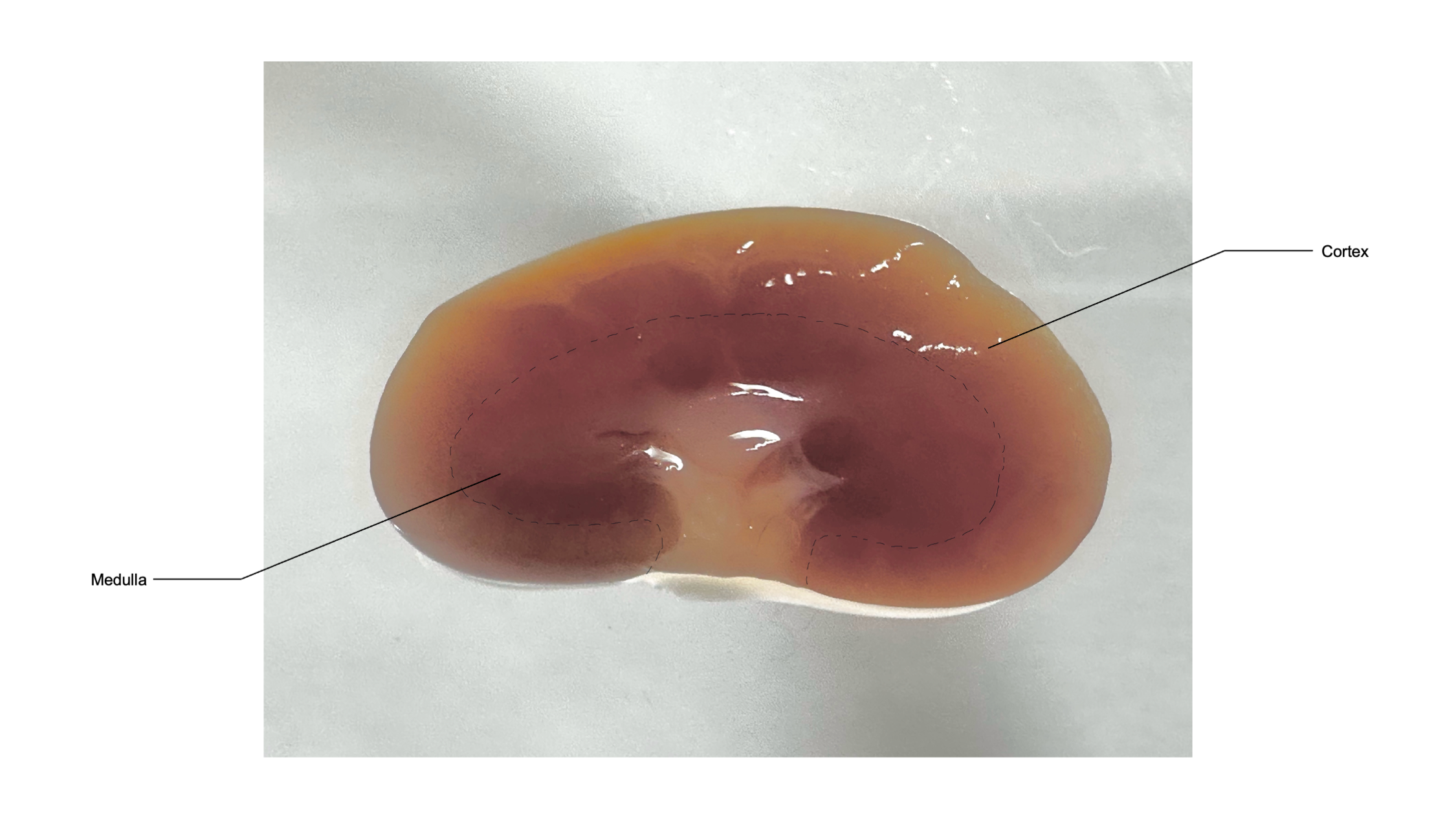

Supplement: S1 Fig — The photograph shows the left kidney, ligated and removed after euthanasia, and then split in half, showing a transverse half section. Each kidney was divided using a scalpel at the line separating the cortex and medulla under translucent light and then cryopreserved. (TIFF) [file pone.0328721.s001.tiff]

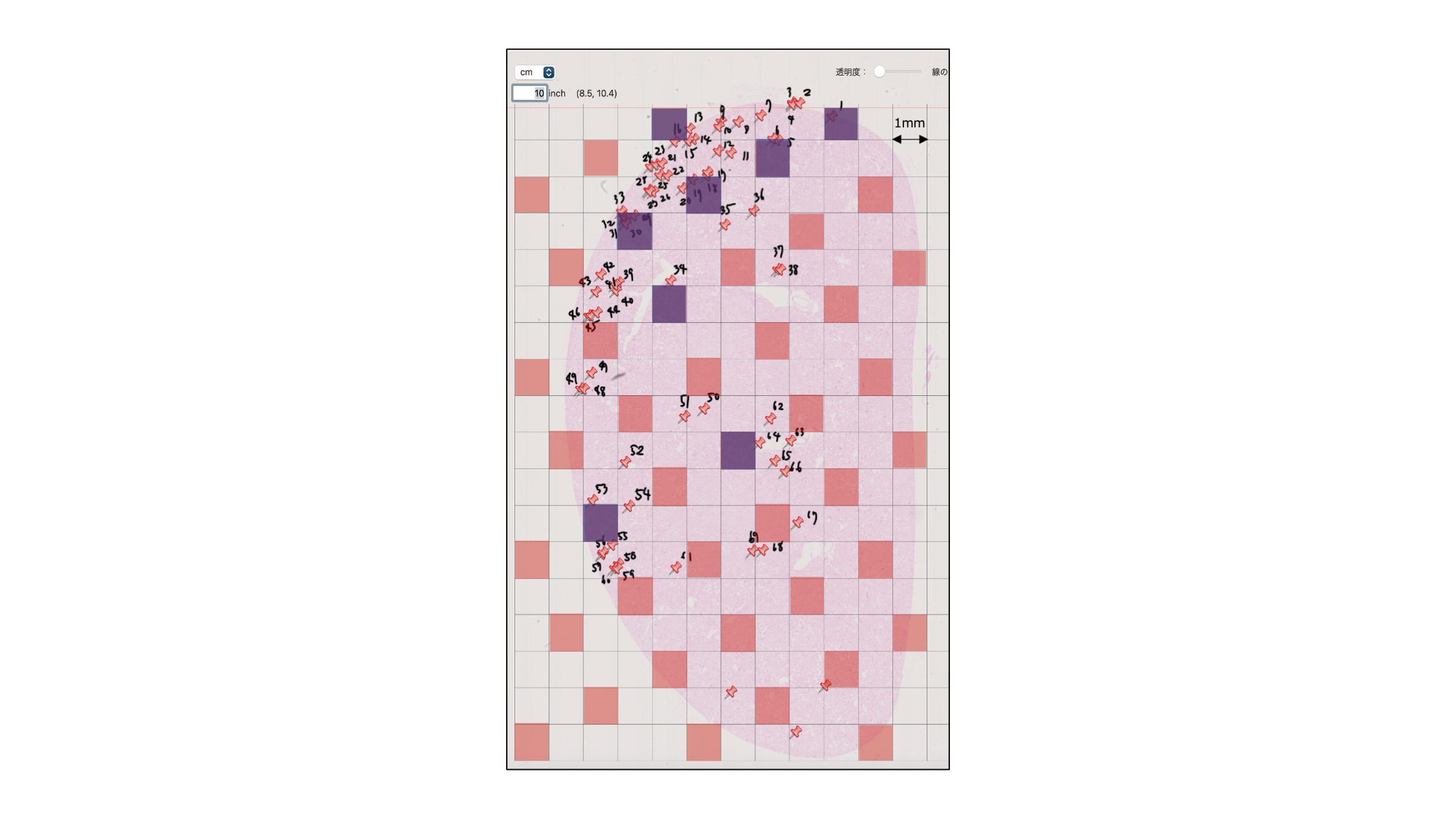

Supplement: S2 Fig — Serial sections of the EG + VitD group in which crystals had formed were stained with Pizzolato stain, and all the crystals that could be identified were plotted. A 1 mm2 grid was then applied, and the crystal-forming tubule segments were counted within the randomly selected area of the grid. (TIFF) [file pone.0328721.s002.tiff]

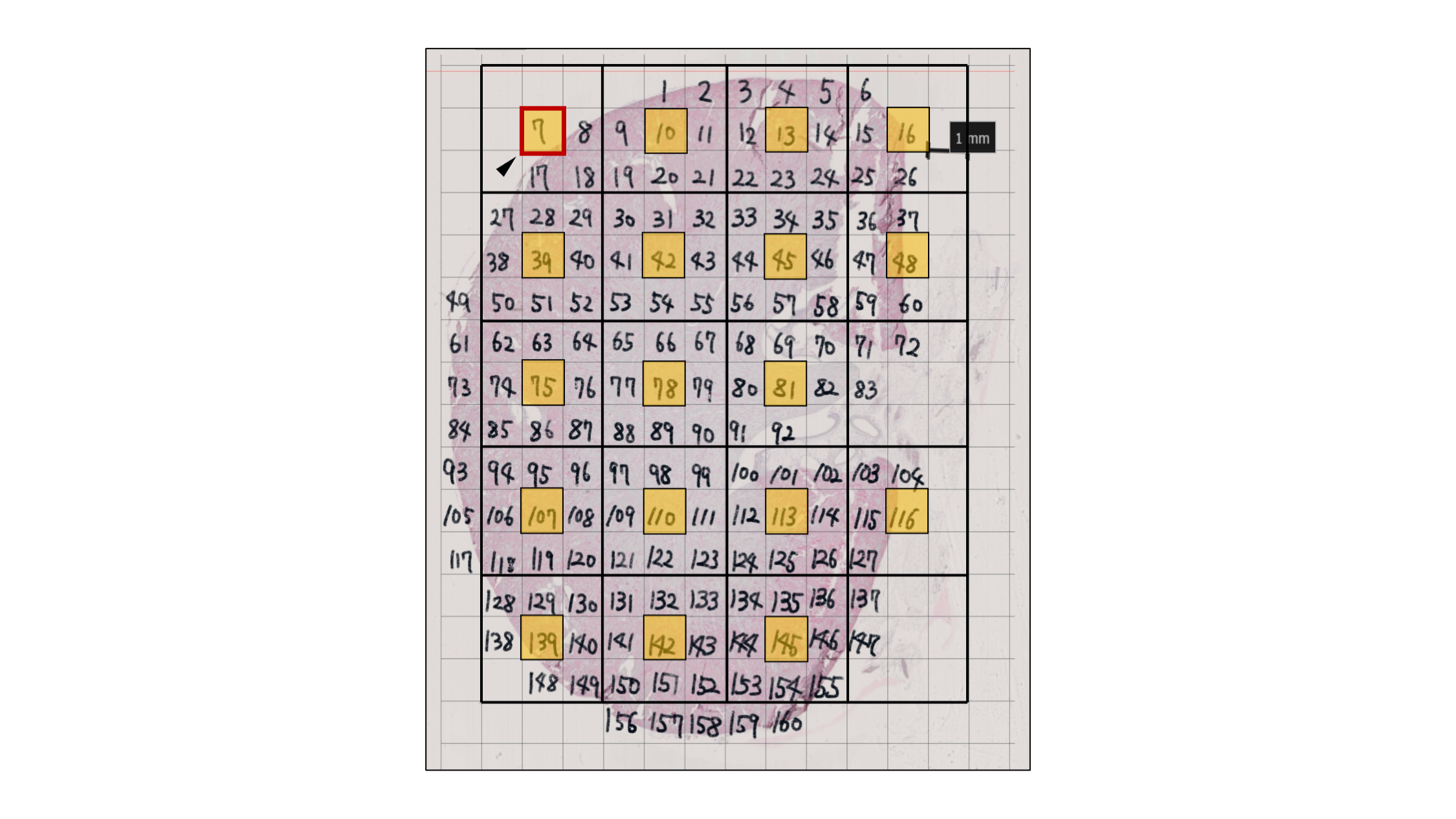

Supplement: S3 Fig — EG + VitD kidney sections double-stained with aquaporin1 and calbindin were fitted to a 1 mm2 grid, and the area corresponding to the central 1 mm2 box was selected as the measurement site in the 9 mm2 box area. (TIFF) [file pone.0328721.s003.tiff]

## Slide 1
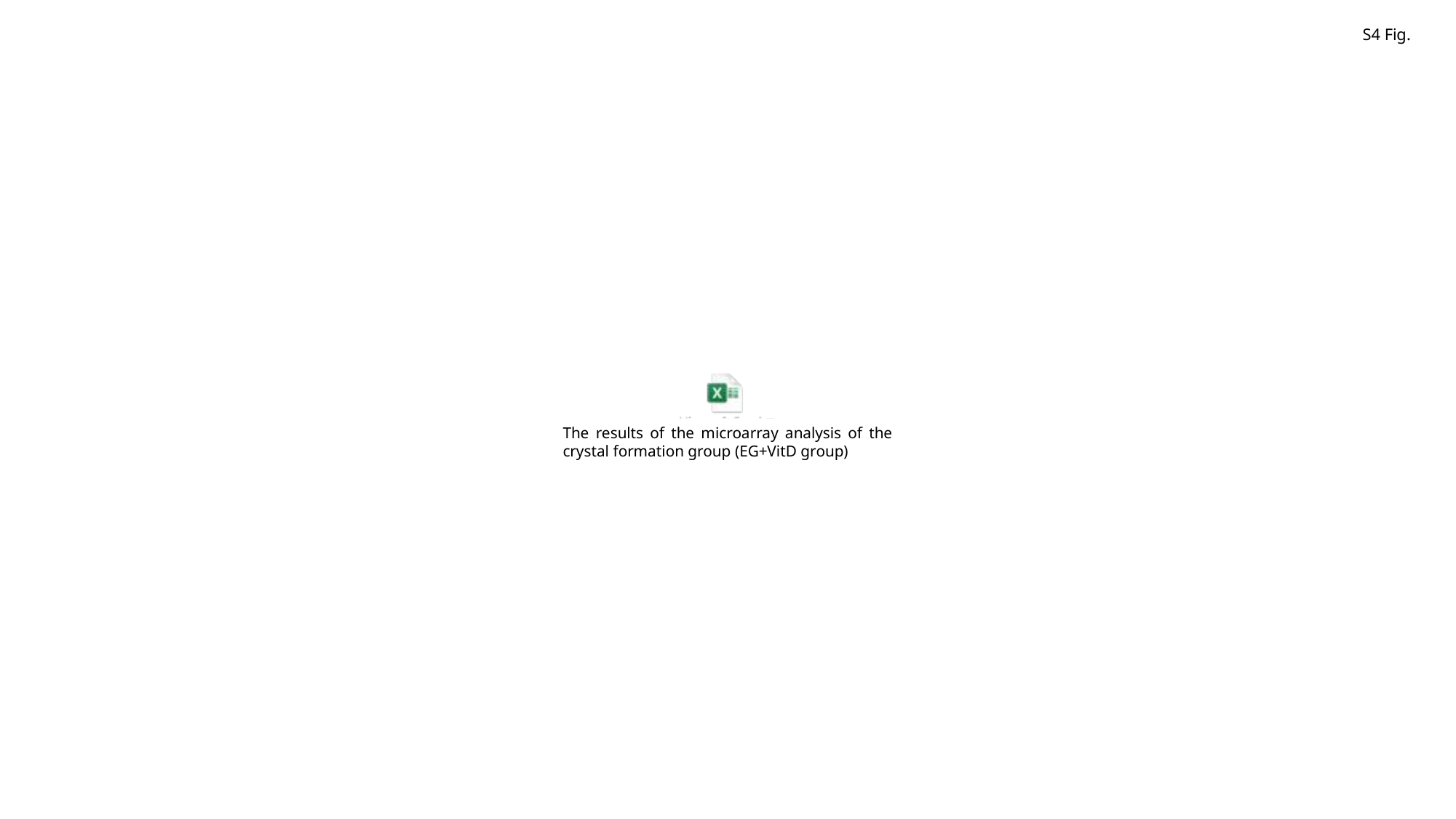

S4 Fig.
The results of the microarray analysis of the crystal formation group (EG+VitD group)

Supplement: S4 Fig — The results of the microarray analysis of the crystal formation group (EG + VitD group) are published as supplementary data. In addition, all microarray data from this study are available under GSE269407 in NCBI-GEO. (PPTX) [file pone.0328721.s004.pptx]

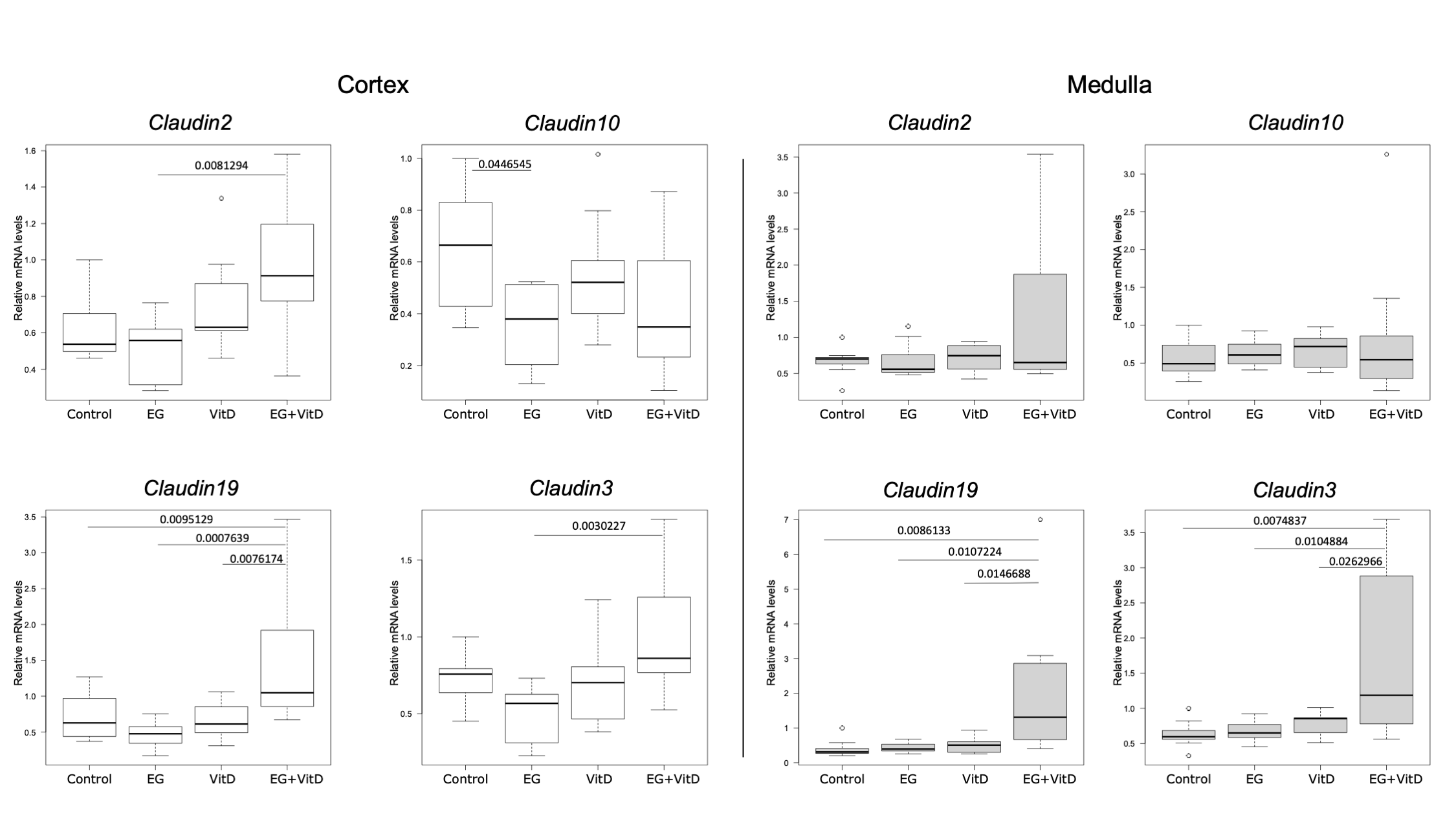

Supplement: S5 Fig — Results of real-time PCR for Claudin genes (Claudin2, Claudin10, Claudin3, Claudin19) involved in the regulation of Ca2+ in the renal tubules. The gene expression levels were normalized to those of the housekeeping gene (18S rRNA) and the data are expressed as absolute quantification values using the standard curve method. Statistical differences between groups were calculated using the Tukey-Kramer method and one-way analysis of variance (ANOVA). Values are presented as the mean ± SD. *P < 0.05 (N = 8). (TIFF) [file pone.0328721.s005.tiff]
